# The Effects of Dietary Orange Peel Fragments Enriched with Zinc and Vitamins C and E on the Antioxidant and Immune Responses of Nile Tilapia under Stress Conditions

**DOI:** 10.3390/ani14202962

**Published:** 2024-10-14

**Authors:** Igor Simões Tiagua Vicente, Luciana Francisco Fleuri, William dos Santos Xavier, Matheus Gardim Guimarães, Pedro Luiz Pucci Figueiredo de Carvalho, Edgar Junio Damasceno Rodrigues, Carlos Eduardo Fonseca Alves, Aline Nunes, Giuseppina Pace Pereira Lima, Samir Moura Kadri, Luiz Edivaldo Pezzato, Margarida Maria Barros

**Affiliations:** 1 Department of Breeding and Animal Nutrition, School of Veterinary Medicine and Animal Science, São Paulo State University (UNESP), Botucatu 18618-681, Brazil; william.xavier@unesp.br (W.d.S.X.); m.guimaraes@unesp.br (M.G.G.); e.rodrigues@unesp.br (E.J.D.R.); luiz.pezzato@unesp.br (L.E.P.); margarida.barros@unesp.br (M.M.B.); 2 Department of Chemistry and Biochemistry, Institute of Biosciences, São Paulo State University (UNESP), Botucatu 18618-681, Brazil; luciana.fleuri@unesp.br (L.F.F.); alinenunes_bio@hotmail.com (A.N.); pace.lima@unesp.br (G.P.P.L.); 3 Department of Ecology and Conservation Biology, Texas A&M University System, College Station, TX 77843, USA; pedro.puccifigueired@agnet.tamu.edu; 4 College of Veterinary Medicine and Animal Sciences, São Paulo State University (UNESP), Botucatu 18618-681, Brazil; carlos.e.alves@unesp.br (C.E.F.A.); samir.kadri@unesp.br (S.M.K.)

**Keywords:** antioxidant enzyme activity, bacterial challenge, hematological profile, *Oreochromis niloticus*, oxidative stress, thermal stress, transport-induced stress

## Abstract

**Simple Summary:**

Aquaculture production has recently surpassed wild fish capture, a growth largely driven by the intensification of fish farming. However, this rapid expansion increases the risk of disease outbreaks and impairs growth performance. These adverse effects can be mitigated through the use of functional additives, such as minerals, vitamins, and industry byproducts with immunomodulatory properties. In this context, we evaluated the effects of functional diets supplemented with vitamins C and E, zinc, and orange peel fragments on the health status and growth performance of juvenile Nile tilapia under stress conditions. The results of this study showed that orange peel fragments, in combination with vitamins C and E, and zinc, produced synergic effects on the immune and antioxidant responses of Nile tilapia subjected to stress. These findings suggest that this supplementation could be an effective nutritional strategy to mitigate the adverse effects of intensive fish farming.

**Abstract:**

This study aimed to evaluate the effect of dietary orange peel fragments (OPFs) enriched with vitamins C (C) and E (E), as well as zinc (Zn) on the growth performance, hematological profile, immunological parameters, antioxidant capacity, and fillet lipid peroxidation of Nile tilapia subjected to heat/dissolved oxygen-induced stress (HDOIS), transport-induced stress (TIS), and *Aeromonas hydrophila* infection (BC). A group of 500 male Nile tilapia (2.7 ± 0.03 g) was randomly distributed in twenty-five 250 L aquaria (20 fish/aquarium) and fed diets containing OPFs (6 g kg^−1^), OPFs/C (6 g kg^−1^/1.8 g kg^−1^), OPFs/E (6 g kg^−1^/0.4 g kg^−1^), OPFs/Zn (6 g kg^−1^/0.21 g kg^−1^), or OPFs/C/E/Zn (6 g kg^−1^/1.8 g kg^−1^/0.4 g kg^−1^/0.21 g kg^−1^) for 100 days. The diets were formulated to contain 30% crude protein and 17 MJ kg^−1^ gross energy. After the feeding period, three groups of fish were independently subjected to a different type of stress: HDOIS (34 °C) for two days; TIS for four hours, or BC for 15 days. The hematological profile, antioxidant capacity, and fillet lipid peroxidation were determined before and after all the stress treatments, along with immunological parameters, which were investigated only for the fish subjected to bacterial infection. In summary, the results showed that growth was not affected by the OPFs, nor by the OPFs enriched with C, E, and Zn; bacterial infection determined anemia for the fish fed any of the experimental diets; the OPFs did not prevent lipid peroxidation under TIS and BC; on the other hand, when enriched with C/E/Zn, lipid peroxidation decreased under HDOIS and TIS. In conclusion, the OPFs enriched with C/E/Zn showed a synergistic effect that promoted an increase in antioxidant enzyme activity, a decrease in lipid peroxidation, and the maintenance of the hematological profile under HDOIS and TIS, but they were not able to maintain the health status under BC.

## 1. Introduction

Stress generated by aquaculture intensification may impair the growth and affect the antioxidant and immune systems of fish, leading to economic losses and creating bottlenecks in aquaculture production globally [1]. To prevent and reduce mortality, it is imperative that fish be provided with diets that attend to their nutritional requirements, while minimizing stress during production. Therefore, the aquafeed industry has been focusing its efforts on functional diets using suitable feedstuffs and additives that meet the fishes’ requirements under the challenging conditions caused by intensive culture systems [2].

The study of functional feedstuffs is a timely branch of aquaculture research, in which the utilization of ingredients such as orange peel fragments (OPFs) could be considered a nutritional strategy to strengthen the fishes’ immune systems and enhance their antioxidant capacity [3,4,5]. The annual citrus fruit production of 100 million tons annually makes it the world’s largest fruit crop [6,7]. Brazil is the biggest producer worldwide, with the forecast of 16,500,000 million tons for 2022 [8]. The global processing of this agro-industrial residue, which includes husks and seeds, generates 60 million tons of waste annually [9], which is costly to dispose of and can be harmful to the environment [10].

Orange peel fragments, which correspond to the outer part of the orange’s epicarp, is an interesting agro-industrial residue [11] that is enriched with important bioactive compounds, such as flavonoids. These polyphenols can modulate immune responses by increasing immune cell activity and triggering the proteins and receptors involved in phagocytosis signaling and microbial destruction [12,13,14,15]. However, flavonoids are widely known for their capacity to modulate antioxidant enzyme activity, such as superoxide dismutase, catalase, and glutathione peroxidase, thereby mitigating oxidative stress and preventing oxidative damage to DNA, proteins, and lipids [3,14,16].

In addition to flavonoids, the minerals and vitamins contained in the premixes that are usually included in aquatic feeds also contribute to the maintenance of the redox status of fish [17]. Zinc (Zn), a trace element, not only affects leukocyte activity, enhancing macrophage chemotaxis and improving disease resistance, but also acts as a cofactor of superoxide dismutase enzyme, thus preventing oxidative stress [18]. Furthermore, vitamins possess the ability to directly neutralize reactive oxygen species (ROS). The vitamins C (C) and E (E) exhibit potent oxidative and reducing proprieties, respectively, thereby preventing lipid peroxidation and reducing oxidation when acting synergistically [18]. Vitamin C also supports the redox recycling of α-tocopherol, which is involved in the reduction of free radicals, thus protecting against the potentially deleterious reactions of reactive oxidizing species [18].

Recent approaches to different stress conditions in fish production have shown that the addition of vitamins, minerals, and bioactive compounds to diets is a valuable nutritional strategy to minimize the consequences of stress. Therefore, considering the importance of OPFs in the aquatic feed industry and the results of a previous study that defined an appropriate dietary inclusion level under specific conditions [3], this study aimed to improve OPFs’ antioxidant and immune capacity by associating this functional ingredient with important vitamins and minerals. Thus, the synergism of OPFs, C, E, and Zn on the growth performance, hematological profile, immunological parameters, antioxidant enzyme activity, and lipid peroxidation of Nile tilapia subjected to different stresses was analyzed.

## 2. Materials and Methods

This study comprised two phases. In Phase I (100 days), the fish were fed experimental diets supplemented with OPFs, C, E, and Zn to improve their nutritional status and stress tolerance. In Phase II, different groups of fish were subjected to three stressful conditions: heat/dissolved oxygen-induced stress, transport-induced stress, and bacterial challenge. The hematological profile, antioxidant enzyme activity, and immunological parameters were evaluated, and the values obtained before and after stress were compared to determine the potential synergistic effects of OPFs, vitamins, and Zn.

### 2.1. Experimental Diets (Phases I and II)

Five practical diets including OPFs, C, E, and Zn supplementation were prepared as follows:

OPFs (6 g kg^−1^ OPFs);
OPFs/C (6 g kg^−1^/1.8 g kg^−1^);
OPFs/E (6 g kg^−1^/0.4 g kg^−1^);
OPFs/Zn (6 g kg^−1^/0.21 g kg^−1^);
OPFs/C/E/Zn (6 g kg^−1^/1.8 g kg^−1^/0.4 g kg^−1^/0.21 g kg^−1^).

The dietary supplementation levels were chosen according to the literature: orange peel fragments, [3]; vitamin C, [19]; vitamin E, [20]; and zinc, [21]. These diets were formulated to contain 30% crude protein and 17 MJ kg^−1^ gross energy, meeting the nutritional requirements for the species [22] (Table 1). Soybean meal and corn were used as the main dietary protein and energy sources due to their nutrient balance and quality as well as market availability. The mineral and vitamin premix contained no Zn, C, or E. The vitamin C that was utilized to formulate the experimental diets was obtained from DSM—Animal Nutrition & Health, Kaiseraugst Switzerland (Rovimix^®^ Stay C, 35% activity); the vitamin E was included as α-tocopheryl acetate (Rhoster Animal Nutrition, 50% activity, Araçoiaba da Serra, SP, Brazil); and zinc was supplemented as zinc sulphate monohydrate (Vetec Química Fina, 98%, Rio de Janeiro, RJ, Brazil). The orange peel fragments of sweet orange (*Citrus sinensis* (L.) Osbeck) were obtained from JBT Food Tech (Araraquara, SP, Brazil). The orange peel fragments were dried in a forced-air oven (50 °C), ground, and stored at −20 °C until further use. To prepare the diets, the ingredients were ground (32-mesh Tyler sieve), weighed, and mechanically mixed with water (23% of dry weight) in a KitchenAid multifunctional mixer (Ação Científica, Piracicaba, SP, Brazil), and the extruded pellets were obtained through a single-screw laboratory extruder (Exteec, Ribeirão Preto, SP, Brazil). The diets were air-dried overnight (55 °C) and stored at 5 °C until further use. The chemical composition of the experimental diets and the OPFs were determined according to AOAC protocols [23] (Table 1).

### 2.2. Antioxidant Properties of OPFs and Diets

The free radical scavenging capacity of the OPFs and the diets was evaluated according to [24] and modified by [25]. Briefly, dried samples (500 mg) were weighed in a light-protected centrifuge tube, extracted with ethanol, and mixed with a 40 µM DPPH ethanolic solution. Trolox was used as the reference standard to convert the inhibitory capacity of each sample to the equivalent antioxidant power. The ferric reducing power (FRAP) analysis followed the protocol described by [26]. The principle of this method is based on the reduction of a ferric-tripyridyltriazine complex to its ferrous, colored form in the presence of antioxidants (Table 1).

The total phenolic content was determined according to [27]. Extracts were prepared using an acidified methanol solution. Folin–Ciocalteu reagent and 20% sodium carbonate were added to the mixture. The results were calculated using a gallic acid calibration curve, and the absorbance was measured at 725 nm. For total flavonoids analysis, extracts were prepared according to [28], with modifications. The extraction was performed using an acidified methanol solution, followed by the addition of an aliquot of 5% aluminum chloride solution. The absorbance was measured at 425 nm (Spectrophotometer BEL Photonics, SP 2000 UV/vis, Thermo Fisher Scientific, Waltham, MA, USA). The total flavonoid content was calculated using a quercetin standard curve, and the results were presented as the mg 100 g^−1^ quercetin equivalent of the dry mass (Table 1).

### 2.3. Feeding Trial (Phase I)

A group of 500 male Nile tilapia fingerlings was obtained from a commercial fish farm (Palmital, SP, Brazil) and transferred to the AquaNutri Laboratory facilities (FMVZ, Botucatu, SP, Brazil). During the adaptation period (30 days), the fish were fed a practical diet (0 OPFs), as presented by [3].

**Figure 1:**
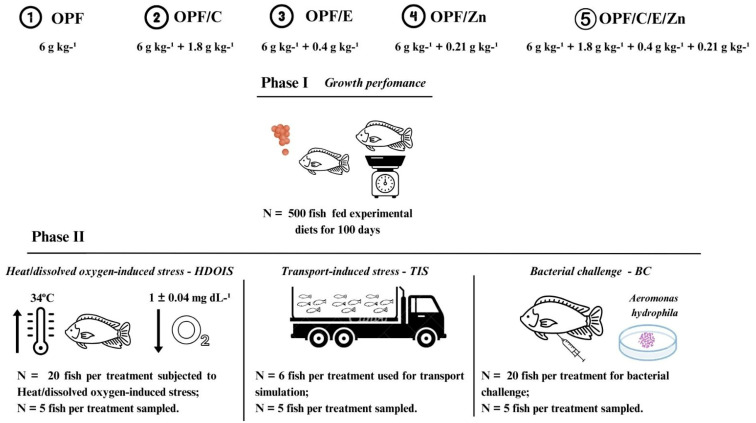
A group of 500 male Nile tilapia fingerlings were randomly sampled (2.7 ± 0.03 g, mean ± SD), stocked in twenty-five 250 L aquaria (20 fish/aquarium), and fed the experimental diets for 100 days. After the feeding trial, growth performance was calculated. After 100 days of feeding and before the fish were weighed, five fish per treatment (first group) were anesthetized to determine the hematological profile, antioxidant capacity, and fillet lipid peroxidation level (before stresses). Subsequently, a 100-fish group was randomly chosen from each aquarium (20 fish/treatment^−1^), transferred to a challenge room, and subjected to HDOIS. After 48 h, the aforementioned analyses were performed. Another group of 30 fish was randomly chosen from each aquarium (six fish/treatment^−1^) and then subjected to TIS, and after 4 h, the analyses were performed. Finally, another group of 100 fish was randomly chosen from each aquarium (20 fish/treatment^−1^) and infected with *Aeromonas hydrophila*, and after 15 days, the same analyses, along with the immunological parameters, were performed.

Subsequently, the fish were randomly sampled (2.7 ± 0.03 g, mean ± SD), stocked in twenty-five 250 L aquaria (20 fish/aquarium), and fed the experimental diets for 100 days. Each aquarium was considered as an experimental unit (five aquaria/treatment) (Figure 1). The fish were hand-fed four times daily until apparent satiation. The fish in each experimental unit were offered small amounts of feed, and their feeding activity was observed. The feed was gradually offered until the majority of the fish began to slow down or stop eating, thus avoiding uneaten pellets. The recirculated system was supplied with a 12 h light:12 h dark photoperiod, and water quality parameters were recorded once a week using a YSU 556 multi-probe system (YSI Environmental, Yellow Springs, OH, USA). The water temperature was heat-controlled at 26 ± 0.5 °C, whilst the dissolved oxygen and pH levels were kept at 6.7 ± 0.25 mg L^−1^ and 6.2 ± 0.23, respectively.

After the 100-day feeding trial, the growth performance was calculated as follows:

Weight gain (WG) = final body weight (g) − initial body weight (g).
Feed intake (FI) = dry feed intake (g).
Feed efficiency (FE) = weight gain (g)/dry feed intake (g).
Specific growth rate (SGR) = (Ln of final weight − Ln of initial weight) × 100/experimental period.
Survival rate (SUR) = (initial fish number − final fish number)/initial fish number × 100 (%).

### 2.4. Stresses and Analyses

After 100 days of feeding and before the fish were weighed, five fish per treatment (first group) were anesthetized (benzocaine, 0.1 g L^−1^) to determine the hematological profile, antioxidant capacity, and fillet lipid peroxidation level (before stress). Subsequently, a 100-fish group was randomly chosen from each aquarium (20 fish/treatment^−1^) and transferred to a challenge room and subjected to HDOIS. After 48 h, the aforementioned analyses were performed. Another group of 30 fish was randomly chosen from each aquarium (six fish/treatment^−1^) and then subjected to TIS, and after 4 h, the analyses were performed. Finally, another group of 100 fish was randomly chosen from each aquarium (20 fish/treatment^−1^) and infected with *Aeromonas hydrophila*, and after 15 days, the same analyses, along with the immunological parameters, were performed. For each stress, a comparison between before and after the stress conditions was assessed to better understand whether the fish were nutritionally prepared to endure the induced stress, and each sampled fish was considered as an experimental unit (Figure 1).

### 2.5. Heat/Dissolved Oxygen-Induced Stress (HDOIS) (Phase II)

For HDOIS, 100 fish were transferred to a challenge room with twenty-five 40 L plastic aquaria with filter systems, individual aeration, and thermostat-controlled heaters to increase the water temperature. The aquaria were divided into four sections with plastic net dividers to avoid agonistic interactions [29]. The fish were randomly distributed at a density of four fish per aquarium and subjected to heat/dissolved oxygen-induced stress by gradually increasing the water temperature, following the methodology described in [3,29]. The thermostat-controlled heaters were set to increase the initial temperature (26 °C) by 1 °C h^−1^ until 34 °C. After that, the thermostat-controlled heaters were set to maintain the water temperature at 34 °C until the end of the HDOIS. The fish were subjected to this adverse condition during a 48 h period. During this period, the oxygenation levels dropped from 5.2 ± 0.2 to 1 ± 0.04 mg dL^−1^. The temperature and the dissolved oxygen concentration were monitored hourly, using a YSI 556 multi-probe system (YSI Environmental Yellow Springs, OH, USA). The fish were fed the same experimental diets as in the feeding trial. At the end of HDOIS, the fish were anesthetized (benzocaine, 0.1 g L^−1^) and euthanized (benzocaine, 1 g L^−1^), and analyses were conducted (five fish/treatment).

### 2.6. Transport-Induced Stress (TIS) (Phase II)

TIS was performed following the methodology described by [30]. Briefly, 30 fish (six fish/treatment) were assigned to five 15 L net cages, which were randomly distributed in a commercial 400 L fish transportation tank, and provided with supplementary aeration (Trevisan, model 50.02.003, Palotina, PR, Brazil). The transport simulation lasted four hours, at night. Every hour, the temperature and the dissolved oxygen concentration were monitored using the YSI 556 multi-probe system, and the ammonia concentration was determined using a commercial kit (Alcon, Camboriú, SC, Brazil). After four hours under the transport simulation, the fish (five fish/treatment) were anesthetized (benzocaine, 0.1 g L^−1^) and euthanized (benzocaine, 1 g L^−1^) and sampled for further analyses.

### 2.7. Bacterial Challenge (BC) (Phase II)

For the bacterial challenge, *Aeromonas hydrophila* (Strain T3R 16s ribosomal RNA gene) were cultured in a brain–heart infusion (BHI) medium at 25 °C for 18 h. The bacterial concentration utilized a lethal dose (LD_50_) determination that was based on a previous study. In short, for the LD_50_ determination, seven groups of 16 fish were infected with an intracoelomatic injection of *Aeromonas hydrophila* (10^2^, 10^3^, 10^4^, 10^5^, 10^6^, 10^7^, and 10^8^ CFU mL^−1^); another 16 fish were injected with 0.9% saline solution to fulfill the role of a negative control. The fish mortality rate was recorded for 15 days. After that, the LD_50_ was calculated following the methodology described by [31]. After the LD_50_ assessment, 100 fish (82.48 ± 2.16 g; twenty fish/treatment) were intraperitoneally (IP) injected with 100 µL of *Aeromonas hydrophila* solution (10^4^ CFU) and randomly distributed into twenty-five 40 L aquaria in an independent system. The mortality was recorded for 15 days, and the fish (five fish/treatment) were then anesthetized (benzocaine, 0.1 g L^−1^) and euthanized (benzocaine, 1 g L^−1^) for sampling and further analysis.

### 2.8. Hematological Assay (Phases I and II)

After the fish (five fish/treatment) were randomly collected and anesthetized (benzocaine, 0.1 g L^−1^), blood was drawn from the caudal vein using a tuberculin syringe that was rinsed with anticoagulant (3% EDTA, Vetec, Química Fina Ltda, Duque de Caxias, RJ, Brazil) for the determination of the red blood cell (RBC), hematocrit (Htc), and hemoglobin (Hb) levels. The RBC count was determined by dilution and enumeration using a hemocytometer. The Htc level was determined using the microhematocrit method, as described by [32]. The Hb level was determined by the cyanmethemoglobin colorimetric method, using a commercial kit (Gold Analisa Diagnóstica, Belo Horizonte, Brazil), according to [33]. The mean corpuscular volume [MCV = (Htc × 10)/erythrocytes] and the mean corpuscular hemoglobin concentration [MCHC = (Hb × 100)/Htc] were calculated according to [34].

### 2.9. Immunological Assay (Phases I and II)

Leukocytes were isolated from the head- and trunk-kidneys of three fish per treatment, as described by [35]. The samples were obtained from the same fish that had been used for the hematological profile determination before and after stress. The head- and trunk-kidney tissues were stored in an L-15 medium (2% fetal calf serum) and homogenized using a glass Potter–Elvehjem tissue grinder. The homogenized tissue was filtered through a 100 µm sterile nylon mesh, and the resulting cell suspension was centrifuged and washed with cold sterile phosphate buffer saline (PBS). The isolated cells were layered on a Percoll gradient (51% *v*/*v*) and centrifuged at 400× *g* for 30 min. The cell layer at the interface was collected and washed twice with an ice-cold PBS at 200× *g* for 10 min. The leukocytes were enumerated using a hemocytometer, and the viability was assessed by Trypan blue staining. The samples were considered adequate when cell survivability was greater than 95%. The cell suspensions were adjusted in an L-15 medium (0.1% fetal bovine serum), and 1.0 mL of the leukocyte suspension was added per well to a sterile, flat-bottomed, 24-well microplate. The microplates were incubated at 27 °C for 2 h to allow for cell attachment.

After incubation, the supernatant was collected for nitric oxide (NO) measurement. Hydrogen peroxide (H_2_O_2_) was measured in the cell monolayer, where most of the cells were macrophages. The NO concentration in the supernatant was measured using the Griess reaction [36]. Different concentrations of the NaNO_2_ solution were used to prepare a standard curve, and readings were taken using a spectrophotometer (Spectrophotometer Thermo Scientific, Evolution 60S UV/vis, Thermo Fisher Scientific, Waltham, MA, USA) at 540 nm. The values were expressed as µmol 10^−5^ cells. The hydrogen peroxide production was measured using the phenol red oxidation method [37]. A standard curve was prepared using different concentrations of H_2_O_2,_ and readings were taken (Spectrophotometer Thermo Scientific, Evolution 60S UV/vis) at 620 nm. The H_2_O_2_ concentration values were expressed as nmol 10^−5^ cells. The intracellular production of superoxide anion (O_2_^−^) was estimated by the formation of formazan granules, as described by [35]. The absorbance values were read in a plate reader spectrophotometer that was operating at 620 nm.

### 2.10. Antioxidant Enzyme Activity (Phases I and II)

Liver samples were obtained from the same fish that had been used for the hematological and immunological determinations before and after stress. The fish (five fish/treatment) were euthanized with a lethal dose of benzocaine (1 g L^−1^), and liver samples were collected, immediately immersed in liquid nitrogen, and stored at −80 °C until the enzyme activity assessment. Briefly, one gram of liver sample was homogenized in 5 mL of 0.05 M phosphate buffer (pH 7.0), and the homogenate was centrifuged at 5000 rpm for 20 min at 4 °C. The supernatant was used for enzyme activity and total protein content analyses.

Superoxide dismutase (SOD; EC 1.15.1.1) activity was assayed according to [38], who measured that one unit of SOD was required to inhibit 50% nitro blue tetrazolium (NBT). The NBT inhibition was measured spectrophotometrically (Abs), at 560 nm wavelength. The catalase (CAT; EC 1.11.1.6) activity was assayed according to [38]. This protocol is based on the reduction of dichromate (in acetic acid) to chromic acetate in the presence of H_2_O_2_ when heated, forming perchromic acid as an unstable intermediate. The absorbance was measured at 570 nm using a spectrophotometer. The glutathione peroxidase (GPx; EC 1.11.1.9) activity was assayed as described by [39]. The absorbance was measured using a spectrophotometer at 420 nm. For all the antioxidant enzyme assays, the total protein concentration was determined according to [40].

### 2.11. Fillet Lipid Peroxidation (Phases I and II)

Five fish per treatment, before and after stress, were randomly collected and anesthetized (benzocaine, 0.1 g L^−1^) for lipid peroxidation determination, according to [41]. The samples were obtained from the same fish that had been used for the hematological, immunological, and antioxidant enzyme activity determinations before and after stress. Ten grams of fish fillets were homogenized in 50 mL of 7.5% trichloroacetic acid in a Philips mixer for 1 min. The mixture was then filtered, and 5 mL of the extract was transferred to a tube containing 5 mL 2-thiobarbituric acid (0.02 M). The tubes were heated in a boiling-water bath for 30 min and then cooled in an ice-water bath for 10 min. The 2-thiobarbituric acid-reactive substances were measured at 532 nm. The values are expressed as mg malonaldehyde/kg^−1^ (MDA).

### 2.12. Statistical Analysis

The data were expressed as means (n = 5) and the pooled standard deviation (PSD). Prior to statistical analyses, the datasets were tested for normality (Kolmogorov–Smirnov test) and the homogeneity of variance (Bartlett’s test). The data from the growth performance and hematological profiles were subjected to a one-way analysis of variance (ANOVA), followed by Tukey’s test, to determine significant differences among treatments. The effects of HDOIS, TIS, and BC were determined by comparing before and after stress exposure using Student’s *t*-test. The analyses were conducted using Minitab 18.1.1.0. Differences were considered statistically significant at *p* < 0.05. To evaluate *Aeromonas hydrophila* infection, the survival data were analyzed by Kaplan Meyer’s estimator, and the treatments were compared using a Log-Rank test.

A principal component analysis (PCA) was conducted to explore potential groupings based on the stress (HDOIS, TIS, and BC) and the variables (SOD, CAT, GPx, MDA, NO, H_2_O_2_, and O_2_). The singular value decomposition (SVD) algorithm was employed for this analysis, and the results were generated using The Unscrambler^®^ X software (version 10.4). Additionally, average values were utilized for visualizing the data through a graphical representation, where colors were used to denote values via a heatmap. This visualization was created using Microsoft^®^ Excel^®^ spreadsheet software (version 1808).

## 3. Results

### 3.1. Growth Performance

There was no significant difference (*p* > 0.05) in the final body weight, weight gain, feed intake, feed efficiency, specific growth rate, or survival rate (Table 2).

### 3.2. Hematological Parameters before and after Challenges

There was no significant difference (*p* > 0.05) identified among the treatments before or after HDOIS, TIS, and bacterial challenge (*p* > 0.05). By comparing the hematological parameters before and after stress, it was observed that HDOIS decreased the RBC count (*p* < 0.05) in the fish fed OPFs/E. A decrease in the Hb level (*p* < 0.05) was also observed in the fish given the OPFs and the OPFs/C/E/Zn. However, the fish fed the OPFs showed an increase in MCV (*p* < 0.05), after HDOIS (Table 3). The bacterial challenge resulted in a decrease in the Htc level (*p* > 0.05) in the fish fed any of the experimental diets, except for the OPFs/C/E/Zn (Table 3).

### 3.3. Liver Antioxidant Enzyme Activity

**Figure 2:**
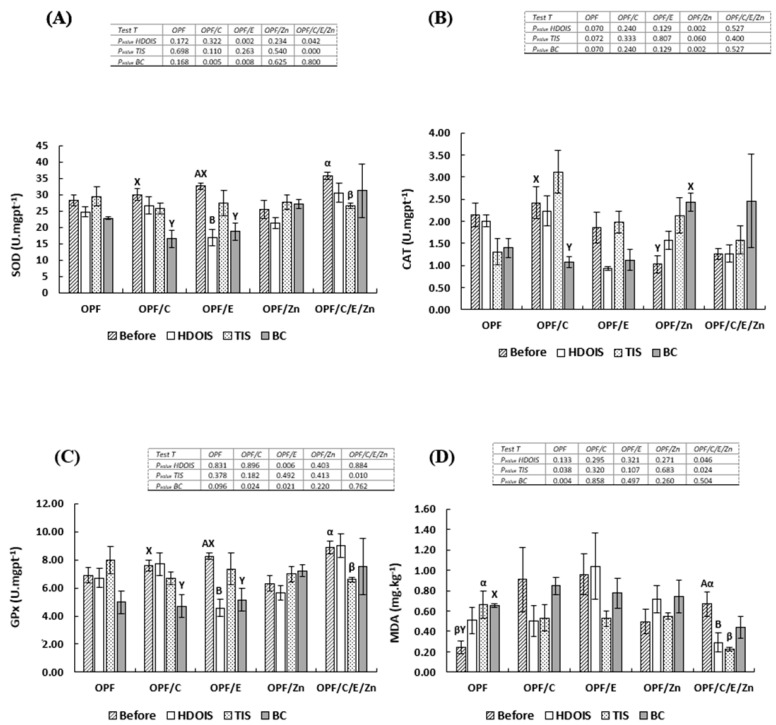
(**A**) Superoxide dismutase activity (SOD), (**B**) catalase activity, (**C**) glutathione peroxidase activity (GPx), (**D**) malondialdehyde concentration (MDA). The data are presented as means ± pooled standard deviation (n = 5). Uppercase letters compare SOD and CAT activities between the moments, before and after stress (*t*-test). A and B uppercase letters compare the hematological response of fish in the same treatment before and after HDOIS;α and β compare before and after; x and y compare before and after BC by a *t*-test (*p* < 0.05); lowercase a and b compare diets among treatments by Tukey’s test (*p* < 0.05). HDOIS, heat/dissolved oxygen-induced stress; TIS, transport-induced stress, and BC, bacterial challenge. OPFs: control 6 g kg^−1^ supplementation of orange peel fragments; OPFs/C: 6 g kg^−1^ supplementation of orange peel fragments and 1.8 g kg^−1^ of vitamin C; OPFs/E: 6 g kg^−1^ supplementation of orange peel fragments and 0.4 g kg^−1^ of vitamin E; OPFs/Zn: 6 g kg^−1^ supplementation of orange peel fragments and 0.2 g kg^−1^ of zinc; OPFs/C/E/Zn: 0.6 g kg^−1^ supplementation of orange peel fragments, 1.8 g kg^−1^ of vitamin C, 0.4 g kg^−1^ of vitamin E, and 0.2 g kg^−1^ of zinc.

By comparing each treatment before and after stress, it was observed that HDOIS determined a decrease (*p* < 0.05) in SOD activity for the fish fed the OPFs/E. In addition, TIS caused a decrease in SOD for the fish fed the OPFs/C/E/Zn diet, and BC promoted a decrease for the fish fed OPFs/C and OPFs/E (Figure 1). As to CAT, the highest activity (*p* < 0.05) was observed after infection when fish were fed the OPFs/Zn, and the lowest for the fish fed OPFs/C (Figure 1). Related to GPx, the fish fed the OPFs/E showed the lowest activity (*p* < 0.05) after HDOIS. After TIS, the lowest activity was observed for the fish given the OPFs/C/E/Zn diet, and after BC the lowest activity was observed for the fish fed OPFs/C and OPFs/E (Figure 2).

### 3.4. Fillet Lipid Peroxidation

By comparing lipid peroxidation before and after stress, the fish fed the OPFs/C/E/Zn showed a decrease after HDOIS and TIS (*p* < 0.05). Regarding TIS and *Aeromonas hydrophila* infection, by comparing the malondialdehyde fillet concentration before and after stress, an increase in lipid peroxidation (*p* < 0.05) in the fish fed the OPFs was identified (Figure 2).

### 3.5. Immunological Parameters and Survival

**Figure 3:**
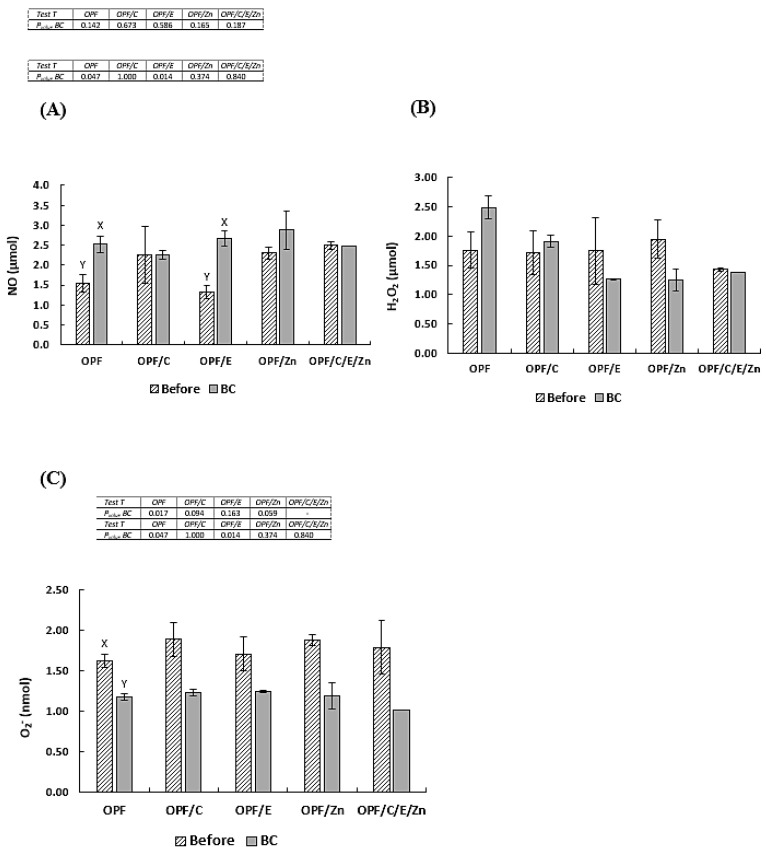
(**A**) Nitric oxide (NO), (**B**) hydrogen peroxide (H_2_O_2_), and (**C**) anion superoxide values are means ± pooled standard deviation (n = 5). Uppercase letters compare NO, H_2_O_2_, and O_2_^−^ concentrations between the moments, before and after stress (*t*-test). HDOIS, heat/dissolved oxygen-induced stress; TIS, transport-induced stress, and BC, bacterial challenge. OPFs: control 6 g kg^−1^ supplementation of orange peel fragments; OPFs/C: 6 g kg^−1^ supplementation of orange peel fragments and 1.8 g kg^−1^ of vitamin C; OPFs/E: 6 g kg^−1^ supplementation of orange peel fragments and 0.4 g kg^−1^ of vitamin E; OPFs/Zn: 6 g kg^−1^ supplementation of orange peel fragments and 0.2 g kg^−1^ of zinc; OPFs/C/E/Zn: 0.6 g kg^−1^ supplementation of orange peel fragments, 1.8 g kg^−1^ of vitamin C, 0.4 g kg^−1^ of vitamin E, and 0.2 g kg^−1^ of zinc.

**Figure 4:**
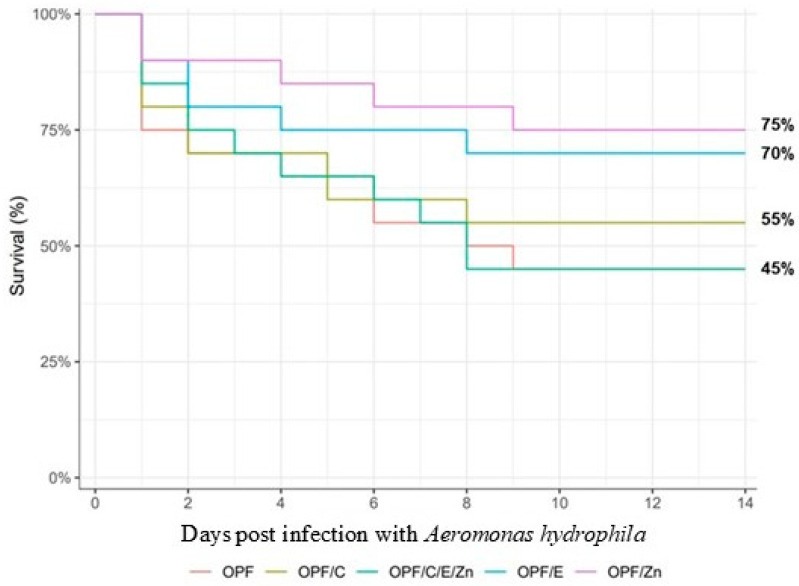
Bacterial challenge assay of Nile tilapia with *Aeromonas hydrophila* after 100 days feeding trial. Kaplan–Meier survival curve of Nile tilapia that were previously fed experimental diets in response to their subsequent infection with *A. hydrophila* for 15 days (Kaplan–Meier survival analysis *p* < 0.05). OPFs: control 6 g kg^−1^ supplementation of orange peel fragments; OPFs/C: 6 g kg ^−1^ supplementation of orange peel fragments and 1.8 g kg^−1^ of vitamin C; OPFs/E: 6 g kg^−1^ supplementation of orange peel fragments and 0.4 g kg^−1^ of vitamin E; OPFs/Zn: 6 g kg^−1^ supplementation of orange peel fragments and 0.2 g kg^−1^ of zinc; OPFs/C/E/Zn: 0.6 g kg^−1^ supplementation of orange peel fragments, 1.8 g kg^−1^ of vitamin C, 0.4 g kg^−1^ of vitamin E, and 0.2 g kg^−1^ of zinc.

The comparison of the data from each treatment before and after *Aeromonas hydrophila* infection showed that the fish given the OPFs or the OPFs/E presented an increase in NO (*p* < 0.05) (Figure 3). The fish fed the OPFs showed a lower O_2_^−^ concentration after BC. After the bacterial challenge, the survival rate was not significantly different (Figure 4).

### 3.6. Principal Component Analysis (PCA) and Heatmap

**Figure 5:**
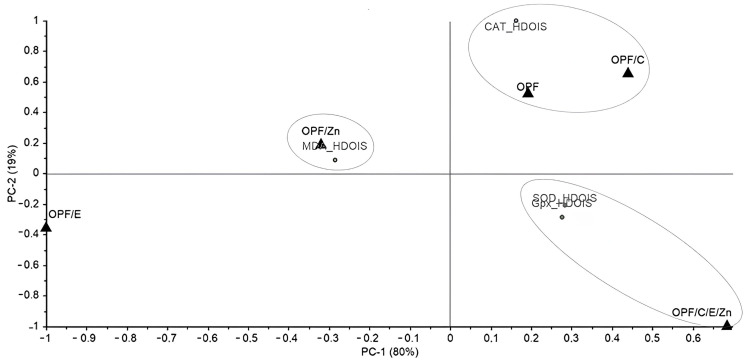
Scatter plot and loadings of principal component analysis (PCA) determined from results obtained by providing different diets (OPFs, OPFs/C, OPFs/E, OPFs/Zn, and OPFs/C/E/Zn) offered to Nile tilapia under heat dissolved oxygen-induced (HDOIS) stress. OPFs: control 6 g kg^−1^ supplementation of orange peel fragments; OPFs/C: 6 g kg ^−1^ supplementation of orange peel fragments and 1.8 g kg^−1^ of vitamin C; OPFs/E: 6 g kg^−1^ supplementation of orange peel fragments and 0.4 g kg^−1^ of vitamin E; OPFs/Zn: 6 g kg^−1^ supplementation of orange peel fragments and 0.2 g kg^−1^ of zinc; OPFs/C/E/Zn: 0.6 g kg^−1^ supplementation of orange peel fragments, 1.8 g kg^−1^ of vitamin C, 0.4 g kg^−1^ of vitamin E, and 0.2 g kg^−1^ of zinc. SOD: superoxide dismutase activity; CAT: catalase activity; GPx: glutathione peroxidase activity; MDA: malondialdehyde concentration; NO: nitric oxide; H_2_O_2_: hydrogen peroxide; O_2_^−^: anion superoxide.

The results were further analyzed using a principal component analysis (PCA) and a heatmap, considering the different types of stress to which the fish were individually subjected (HDOIS, TIS, and BC). For the HDOIS stress, a total data variance of 99% (PC1 and PC2) was observed, with PC1 representing 80% of the model (Figure 5). Three distinct groupings were identified: the OPFs/C and the OPFs treatments (PC1 +, PC2 +) were grouped with the enzymatic activity of catalase (CAT); the OPFs/C/E/Zn treatment (PC1 +, PC2 −) was associated with superoxide dismutase (SOD) and glutathione peroxidase (GPx); and the OPFs/Zn treatment (PC1 −, PC2 +)was clustered with the antioxidant capacity measured by malondialdehyde (MDA). The heatmap (Table 4) confirms that these clusters correspond to the highest values observed for the variables SOD, CAT, and GPx, and with the intermediate value for MDA. Although a higher value for MDA was observed for the OPFs/E compared to the OPFs/Zn, the relationship between the OPFs/E and MDA was not evident, likely due to lower amounts observed in all the other variables compared to the other diets.

**Figure 6:**
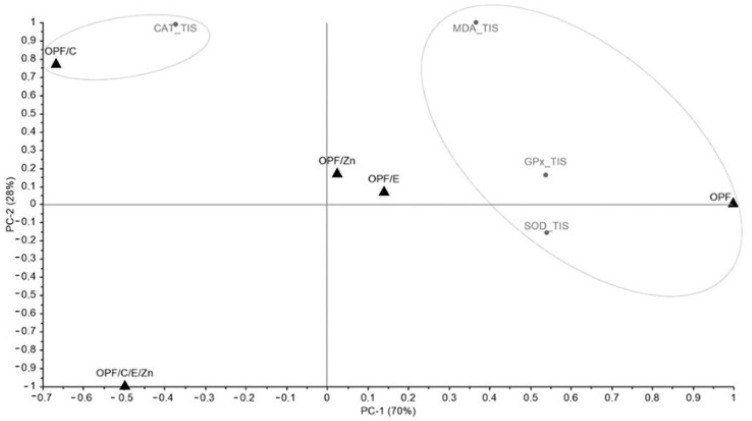
Scatter plot and loadings of principal component analysis (PCA) determined from results obtained by providing different diets (OPFs, OPFs/C, OPFs/E, OPFs/Zn, and OPFs/C/E/Zn) offered to Nile tilapia under transport-induced stress (TIS) stress. OPFs: control 6 g kg^−1^ supplementation of orange peel fragments; OPFs/C: 6 g kg^−1^ supplementation of orange peel fragments and 1.8 g kg^−1^ of vitamin C; OPFs/E: 6 g kg^−1^ supplementation of orange peel fragments and 0.4 g kg^−1^ of vitamin E; OPFs/Zn: 6 g kg^−1^ supplementation of orange peel fragments and 0.2 g kg^−1^ of zinc; OPFs/C/E/Zn: 0.6 g kg^−1^ supplementation of orange peel fragments, 1.8 g kg^−1^ of vitamin C, 0.4 g kg^−1^ of vitamin E, and 0.2 g kg^−1^ of zinc. SOD: superoxide dismutase activity; CAT: catalase activity; GPx: glutathione peroxidase activity; MDA: malondialdehyde concentration.

For the TIS analysis, a total variance of 98% was obtained in the PCA (PC1 and PC2), with PC1 representing 70% of the model (Figure 6). In this PCA, two clusters were identified, with the OPFs diet grouped with MDA, GPx, and SOD (PC1 +, PC2 −, PC2 +), and OPFs/C grouped with CAT (PC1 −, PC2 +). Upon analyzing the heatmap, it is evident that all clusters are associated with the higher values found in these variables with their respective diets (Table 4).

**Figure 7:**
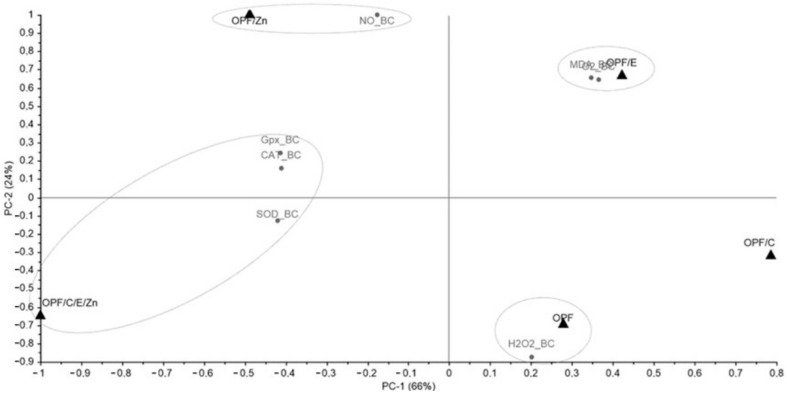
Scatter plot and loadings of principal component analysis (PCA) determined from results obtained by providing different diets (OPFs, OPFs/C, OPFs/E, OPFs/Zn, and OPFs/C/E/Zn) offered to Nile tilapia under *Aeromonas hydrophila* infection (BC). OPFs: control 6 g kg^−1^ supplementation of orange peel fragments; OPFs/C: 6 g kg^−1^ supplementation of orange peel fragments and 1.8 g kg^−1^ of vitamin C; OPFs/E: 6 g kg^−1^ supplementation of orange peel fragments and 0.4 g kg^−1^ of vitamin E; OPFs/Zn: 6 g kg^−1^ supplementation of orange peel fragments and 0.2 g kg^−1^ of zinc; OPFs/C/E/Zn: 0.6 g kg^−1^ supplementation of orange peel fragments, 1.8 g kg^−1^ of vitamin C, 0.4 g kg^−1^ of vitamin E, and 0.2 g kg^−1^ of zinc. SOD: superoxide dismutase activity CAT: catalase activity; GPx: glutathione peroxidase activity; MDA: malondialdehyde concentration; NO: nitric oxide; H_2_O_2_: hydrogen peroxide; O_2_^−^: anion superoxide.

For the BC analysis, the variables nitric oxide (NO), hydrogen peroxide (H_2_O_2_), and superoxide anion (O_2_^−^) were also included in the PCA. The total variance was 90% (PC1 and PC2), with PC1 accounting for 66% (Figure 7). In this case, it was observed that the OPFs/Zn diet was correlated with NO (PC1 −, PC2 +), while the OPFs/E diet was grouped with MDA and O_2_^−^ (PC1 +, PC2 +). The OPFs diet was associated with H_2_O_2_ (PC1 +, PC2 −), and the OPFs/C/E/Zn was most closely related to GPx, CAT, and SOD (PC1 −, PC2 −, PC2 +). From the heatmap, it is evident that the clusters formed are once again related to the highest values observed in the variables, except MDA, which was the second highest for the OPFs/E (Table 4). This observation may be linked to the OPFs/C result, which presented the highest MDA value, but had four variables with lower values than the other diets.

## 4. Discussion

The purpose of this study was to evaluate the improvement of OPFs’ antioxidant and immune capacity by combining this functional ingredient with vitamin C, vitamin E, and zinc, thus investigating such capacity with Nile tilapia under different stress conditions. Overall, C/E/Zn improved OPFs’ functional characteristics and prepared the Nile tilapia to cope with HDOIS and TIS, but not with bacterial infection.

Although the main characteristics of OPFs are their antioxidant and immune capacities [3,42], growth performance was not affected after the 100-day feeding period under the experimental nutritional and environmental conditions, as described as the first phase of the trial. A positive effect of orange peel on Nile tilapia feed intake was previously described by [3,43]. Different from [3], an improvement in SGR, WG, and FCR (2 g kg^−1^ of orange peel/60 days) was observed by [43].

It is known that oxidative stress is triggered when there is an imbalance between the occurrence of reactive oxygen/nitrogen species and the capability to counteract their action by the antioxidative protection, which could damage the structure of lipids, proteins, and nucleic acids within the cellular compartments [44]. This physiological condition could occur when fish are exposed to different stress factors under intensive production [45,46,47]. As for the nutrients used as an enrichment for OPFs, it has been demonstrated that vitamin C not only scavenges hydroxyl, alkoxyl, and superoxide radical anion, but also reactive nitrogenated species, by forming semidehydroascorbic acid, preventing essential biomolecules oxidative degeneration [48]. Its synergistic effect with vitamin E has also been described, demonstrating that at the lipid–aqueous interphase, ascorbic acid reacts with membrane-bound oxidized tocopheroxyl radical, reducing it and regenerating active tocopherol, which in turn will be able to accomplish its antioxidant roles [49]. As for zinc, it has been reported as a component of superoxide dismutase enzyme, which has a potent effect in the antioxidant protection system [50].

Due to the characteristics of the different stress conditions to which the fish were subjected, alterations in the hematological profile were expected. Under high temperature and low oxygen conditions, an increase in RBC and Hb is required [51] to support appropriate tissue oxygenation, as observed in previous studies with Nile tilapia that were subjected to HDOIS (32 °C/30 h—[52,53]). Overall, an opposite response was observed under HDOIS (34 °C/48 h), which could be explained by the increase in the metabolic rate, leading to a superior increment in reactive oxygen species (ROS) production, potentially resulting in cell hemolysis through lipid peroxidation [54]. Similar impairments in the hematological profile were described by [3] under similar experimental conditions, including facilities and diets.

As to lipid peroxidation, it is important to emphasize that the OPFs enriched with vitamin E showed an interesting result after HDOIS, since enzyme activities significantly decreased. This may have occurred due to vitamin E’s antioxidant capacity to reduce ROS levels, thereby decreasing the substrate available for antioxidant enzymes to be active, as well as the antioxidant activity measured by MDA (Figure 1). In sum, we could assume that the OPFs/E was able to mitigate the free radicals’ effects on the formation of peroxides and superoxide by scavenging ROS-like lipid peroxyl radicals, thus acting as an antioxidant chain breaker. This characteristic has been previously described, showing that the α-tocopherol form is an important lipid-soluble antioxidant that protects membranes from oxidation by reacting with the lipid radicals produced in the lipid peroxidation chain reaction [55,56]. However, its concentration was not enough to maintain the RBC number under HDOIS and, although not significant, there was also a slight increment in fillet MDA. Vitamins C and E work synergically, since vitamin C has the capacity to regenerate and/or conserve vitamin E. Together, these vitamins act as antioxidants, stimulating immune responses and fostering the growth of captive fish [57]. Similarly to our results, [58] observed a decrease in antioxidant enzyme activity after feeding juvenile discus fish (*Symphysodon haraldi*) diets that were supplemented with a combination of vitamin E and vitamin C. The authors observed an increment in the antioxidant capacity alongside a decrease in SOD activity, using dietary vitamin concentrations lower than those supplemented in this study. On the other hand, the OPFs enriched with C/E/Zn were able to decrease fillet lipid peroxidation, probably due to the cumulative antioxidant capacity, but were not able to maintain the Hb levels. Similarly, the OPFs without enrichment were not capable of keeping Hb levels under this thermal stress. Overall, the Nile tilapia under 34 °C for 48 h and low oxygen concentration (1.0 ± 0.04 mg dL^−1^) likely required a higher concentration of vitamin E to support the increased demand for oxygen and minimize the negative effects of oxidative stress on the blood cells.

Another stressful condition that could have altered the hematological profile was TIS, a physical cumulative stress, where fish are subjected to handling, a high loading density, and hypoxia in addition to the transport process itself [59]. However, in our study, the fish were probably nutritionally prepared to cope with the 4 h transportation challenge, and there were no significant changes. Our results corroborate the data obtained by [53]. The authors of that study assessed Nile tilapia’s physiological responses under four different types of stress and concluded that TIS and HDOIS were indeed less demanding when compared to cold-induced stress and size-sorting-induced stress. As for antioxidant enzyme activities, the OPFs enriched with C/E/Zn may have been able to neutralize ROS, leading to a decrease in SOD and GPx activities after TIS. The decrease in lipid peroxidation can be confirmed by the significant decline in fillet MDA concentration after transportation. This effect can be attributed to the antioxidant characteristics of the enrichment nutrients, as previously described under the HDOIS stress condition. On the other hand, the OPFs by themselves were unable to prevent lipid peroxidation, leading to an increase in MDA concentration, as observed in the heatmap and PCA analyses (Table 4 and Figure 5). The OPFs enriched with C/E/Zn presented enhanced antioxidant properties, with a 10.68% increase for flavonoids, 52.6% for DPPH, and 17.96% for FRAP.

The hematological profile is considered an important parameter for assessing fish stress conditions or health status. In our study, *Aeromonas hydrophila* infection caused microcytic hypochromic anemia, as evidenced by the decrease in RBC, Htc, and Hb levels, independently of the functional additive tested. This condition may have occurred due to bacterial infection, which produces virulence factors such as hemolysin, causing hemolytic reactions due to the lysis of red blood cells [60,61]. According to [62], this bacterium causes hemorrhagic septicemia. In our study, fish presented typical clinical signs such as hemorrhage, ascites, hyperemia, ulcers, and liver lesions, as described by [63]. Besides the anemia, it can be observed that *A*. *hydrophila* infection weakened the antioxidant ability of the fish, even for those fed potent antioxidants such as vitamin C and vitamin E. The OPFs by themselves were not able to maintain the fish’s antioxidant response, leading to the occurrence of oxidative stress and lipid peroxidation, thus resulting in high fillet MDA concentrations. As for immune response, a limited effect was observed. The OPFs determined a significant increase in NO and a decrease in O_2_^−^ after *A*. *hydrophila* infection, but no significant increase was observed for H_2_O_2_, even though the concentration was 41.4% higher. The dietary OPFs/E also led to a significant increment in NO. This limited effect could be related to the day after infection, when the blood was collected (14th), a time when the immune response could be diminished. This decrease over time of immune responses was described by [64] in Nile tilapia infected with *Aeromonas hydrophila* and *Photobacterium damselae*. Overall, it can be assumed that the OPFs enriched with C/E/Zn showed an interesting antioxidant-boosting capacity, since their supplementation was able to maintain the Nile tilapia’s enzyme activity after all the types of stress. Accordingly, the C/E/Zn enrichment determined the lowest fillet MDA concentration, as can be seen on the heatmap. However, it was not capable of maintaining fish survivability after bacterial infection, showing similar survival rates when compared to the isolated OPFs treatment, and lower than other treatments, although no significant.

In conclusion, enriching OPFs with antioxidant compounds such as vitamin C, vitamin E, and zinc might be an interesting nutritional strategy to enhance fish’s natural ability to cope with stressful conditions and promote the overall health status in aquaculture species.

## Figures and Tables

**Table 1:** Formulation and analyzed proximate composition of experimental diets.

**Ingredients (g kg^−1^)**	**OPFs**	**OPFs/C**	**OPFs/E**	**OPFs/Zn**	**OPFs/C/E/Zn**	
Soybean meal	591.6	592.4	592.4	592.4	592.7	
Corn	339.3	337.8	338.2	338.4	336.9	
Soybean oil	32	32	32	32	32	
DL-Methionine (98%)	2.6	2.6	2.6	2.6	2.6	
L-Threonine (98.5%)	2.5	2.5	2.5	2.5	2.5	
L-Tryptophan (98%)	0.3	0.3	0.3	0.3	0.3	
Dicalcium phosphate	21.1	21.1	21.1	21.1	21.1	
BHT ^1^	0.2	0.2	0.2	0.2	0.2	
Salt	1	1	1	1	1	
Premix Vit/Min ^2^	1.5	1.5	1.5	1.5	1.5	
Vitamin C ^3^	0.9	1.8	0.9	0.9	1.8	
Vitamin E ^4^	0.1	0.1	0.4	0.1	0.4	
Zinc ^5^	0.05	0.05	0.05	0.21	0.21	
Orange peel fragments ^6^	6	6	6	6	6	
**Analyzed proximate**						
Dry matter (g kg^−1^)	900.8	902.8	880.3	908.7	903.5	
Crude protein (g kg^−1^)	308.6	307.7	309.4	291.9	308.0	
Gross energy (MJ.g^−1^)	17.35	17.22	16.91	17.59	17.50	
Crude fiber (g kg^−1^)	56.9	55.1	58.2	56.0	61.6	
Crude fat (g kg^−1^)	32.5	32.1	29.4	26.1	25.3	
Ash (g kg^−1^)	60.9	59.7	56.4	53.5	56.5	
**Antioxidant analyses and phenolic content**						**OPFsp ^7^**
Phenolics (mg.g^−1^)	146.13	148.03	148.18	147.37	151.29	623.33
Flavonoids (mg·g^−1^)	131.77	138.94	128.93	128.82	145.85	383.84
DPPH ^8^ (µg/g TE g^−1^)	4.01	3.96	3.66	3.21	6.12	124.85
FRAP ^9^ (nmol Fe g^−1^)	4.12	4.34	4.48	4.36	4.86	29.81

^1^ Butylated hydroxytoluene—antioxidant. ^2^ Mineral and vitamin premix: vitamin A = 1,200,000 IU; vitamin D_3_ = 200,000 IU; vitamin K_3_ = 2400 mg; vitamin B_1_ = 4800 mg; vitamin B_2_ = 4800 mg; vitamin B_6_ = 4000 mg; vitamin B_12_ = 4800 mg; folic acid = 1200 mg; calcium pantothenate = 12,000 mg; biotin = 48 mg; choline = 65,000 mg; nicotinic acid = 24,000 mg; Mn = 4.000 mg; I = 20 mg; Co = 2 mg; Cu = 4 mg e Se = 20 mg. ^3^ Vitamin C—Rovimix^®^ Stay, C 35 (DSM Nutritional Products, Switzerland). ^4^ Vitamin E—α-tocopheryl acetate, 50% activity (Rhoster Animal Nutrition, Brazil). ^5^ Zinc sulphate monohydrate, 98% purity (Vetec Química Fina, Brazil). ^6^ Orange peel fragments: dry matter (895.5 g kg^−1^), crude protein (61.4 g kg^−1^), gross energy (17.36 MJ.kg^−1^), crude fiber (146.4 g kg^−1^), crude fat (43.1 g kg^−1^). ^7^ OPFp—orange peel fragments product. ^8^ Antioxidant capacity—measured by the DPPH: radical (2,2-diphenyl-1-picryl-hydrazyl) scavenging activity assay. ^9^ FRAP: ferric reducing antioxidant power.

**Table 2:** Initial body weight (IBW), final body weight (FBW), weight gain (WG), specific growth rate (SGR), feed intake (FI), feed efficiency (FE), and survival (SUR) of Nile tilapia that were fed diets containing orange peel fragments, vitamins C and E, and zinc for 100 days.

	Diets						*p* Value
	OPFs	OPFs/C	OPFs/E	OPFs/Zn	OPFs/C/E/Zn	PSD	
IBW	2.74	2.74	2.75	2.74	2.72	0.03	0.849
FBW (g)	79.44	82.83	84.30	83.65	84.70	9.23	0.901
WG (g)	75.25	79.10	80.28	79.94	80.56	9.06	0.881
SGR (%)	3.73	3.78	3.80	3.79	3.81	0.13	0.899
FI (g)	104.94	96.2	109.29	95.26	106.8	20.66	0.752
FE	0.74	0.84	0.76	0.83	0.70	0.14	0.547
SUR (%)	66.0	74.0	69.0	75.0	69.0	9.54	0.583

Values are means ± PSD (pooled standard deviation) of five replicates. No significant difference at *p* > 0.05 (Tukey Test). OPFs: control 6 g kg^−1^ supplementation of orange peel fragments; OPFs/C: 6 g kg ^−1^ supplementation of orange peel fragments and 1.8 g kg^−1^ of vitamin C; OPFs/E: 6 g kg^−1^ supplementation of orange peel fragments and 0.4 g kg^−1^ of vitamin E; OPFs/Zn: 6 g kg^−1^ supplementation of orange peel fragments and 0.2 g kg^−1^ of zinc; OPFs/C/E/Zn: 0.6 g kg^−1^ supplementation of orange peel fragments, 1.8 g kg^−1^ of vitamin C, 0.4 g kg^−1^ of vitamin E, and 0.2 g kg^−1^ of zinc.

**Table 3:** Hematological parameters of Nile tilapia that were fed diets containing orange peel fragments, vitamins C and E, and zinc and that were subjected to HDOIS, TIS, or BC.

		Diets						
		OPFs	OPFs/C	OPFs/E	OPFs/Zn	OPFs/C/E/Zn	*PSD*	*p* Value
RBC (10^6^ mL^−1^)	Before	2.12 **x**	2.11 **x**	2.17 **Ax**	2.05 **x**	1.88 **x**	0.20	0.251
	HDOIS	1.69	1.91	1.78 **B**	1.88	1.70	0.26	0.586
	***p* value**	0.110	0.248	0.007	0.165	0.181		
	TIS	2.11	2.00	2.09	2.20	2.09	0.26	0.848
	***p* value**	0.944	0.525	0.722	0.920	0.342		
	BC	1.60 **y**	1.56 **y**	1.51 **y**	1.51 **y**	1.35 **y**	0.17	0.505
	***p* value**	0.015	0.013	0.000	0.001	0.004		
Hb (g dL^−1^)	Before	7.56 **Ax**	7.84 **x**	7.05	7.17	7.16 **A**	0.79	0.505
	HDOIS	5.89 **B**	7.09	6.03	7.42	6.13 **B**	1.18	0.197
	***p* value**	0.017	0.197	0.307	0.752	0.032		
	TIS	7.34	7.47	7.65	6.42	7.74	0.84	0.141
	***p* value**	0.44	0.46	0.27	0.29	0.37		
	BC	5.95 **y**	5.90 **y**	5.93	5.51	5.78	1.01	0.955
	***p* value**	0.04	0.011	0.056	0.056	0.27		
Htc (%)	Before	33.1 **x**	33.8 **x**	32.4 **x**	34.1 **x**	34.8	2.45	0.595
	HDOIS	27.2	33.0	32.7	34.0	30.6	3.82	0.075
	***p* value**	0.055	0.623	0.837	0.961	0.149		
	TIS	33.6	34.0	33.5	31.6	34.4	2.87	0.416
	***p* value**	0.680	0.190	0.280	0.310	0.870		
	BC	25.2 **y**	26.0 **y**	26.8 **y**	26.4 **y**	26.0	3.80	0.973
	***p* value**	0.024	0.001	0.002	0.025	0.075		
MCV (fL)	Before	156.26 **B**	161.87	162.20	166.25	185.03	16.44	0.099
	HDOIS	169.77 **A**	173.52	183.54	180.86	179.34	12.16	0.447
	***p* value**	0.035	0.346	0.141	0.105	0.431		
	TIS	161.00	171.70	161.00	157.40	167.10	19.09	0.773
	***p* value**	0.680	0.410	0.930	0.350	0.21		
	BC	158.6	166.2	177.51	164.6	192.34	17.39	0.183
	***p* value**	0.884	0.641	0.247	0.846	0.629		
MCHC (%)	Before	22.92	23.23	21.72	20.10	20.60	1.81	0.129
	HDOIS	21.78	21.50	18.36	21.72	21.77	2.84	0.277
	***p* value**	0.338	0.249	0.234	0.558	0.327		
	TIS	21.84	21.88	22.80	20.46	22.46	1.80	0.328
	***p* value**	0.26	0.33	0.31	0.75	0.52		
	BC	23.76	22.6	22.08	20.73	22.22	1.97	0.241
	***p* value**	0.58	0.661	0.716	0.84	0.051		

Values are means ± PSD (pooled standard deviation). RBC, red blood cell count; Hb, hemoglobin; Htc, hematocrit; MCV, mean corpuscular volume; MCHC, mean corpuscular hemoglobin concentration. A and B uppercase letters compare the hematological response of fish in the same treatment before and after HDOIS; x and y compare before and after BC by a *t*-test (*p* < 0.05); lowercase a and b compare diets among treatments by Tukey’s test (*p* < 0.05). OPFs: control 6 g kg^−1^ supplementation of orange peel fragments; OPFs/C: 6 g kg ^−1^ supplementation of orange peel fragments and 1.8 g kg^−1^ of vitamin C; OPFs/E: 6 g kg^−1^ supplementation of orange peel fragments and 0.4 g kg^−1^ of vitamin E; OPFs/Zn: 6 g kg^−1^ supplementation of orange peel fragments and 0.2 g kg^−1^ of zinc; OPFs/C/E/Zn: 0.6 g kg^−1^ supplementation of orange peel fragments, 1.8 g kg^−1^ of vitamin C, 0.4 g kg^−1^ of vitamin E, and 0.2 g kg^−1^ of zinc.

**Table 4:** Heatmap of average of enzyme activity (U mgpt^−1^), fillet MDA concentration (mg kg^−1^), anion superoxide (nmol), nitric oxide, and hydrogen peroxide (µmol) of Nile tilapia under stress conditions.

Treatment	Variables						
**Before**							
	**SOD**	**CAT**	**GPx**	**MDA**	**NO**	**H_2_O_2_**	**O^−^_2_**
OPFs	28.15	2.15	6.90	0.25	1.54	1.76	1.62
OPFs/C	30.02	2.43	7.57	0.91	2.25	1.72	1.89
OPFs/E	32.63	1.86	8.25	0.96	1.32	1.75	1.71
OPFs/Zn	25.48	1.03	6.31	0.50	2.30	1.95	1.88
OPFs/C/E/Zn	35.87	1.26	8.88	0.67	2.49	1.43	1.80
**HDOIS**							
	**SOD**	**CAT**	**GPx**	**MDA**			
OPFs	24.71	2.01	6.72	0.51			
OPFs/C	26.64	2.23	7.70	0.50			
OPFs/E	16.83	0.94	4.59	1.04			
OPFs/Zn	21.25	1.57	5.64	0.71			
OPFs/C/E/Zn	30.56	1.27	9.02	0.29			
**TIS**							
	**SOD**	**CAT**	**GPx**	**MDA**			
OPFs	29.48	1.31	7.96	0.66			
OPFs/C	25.65	3.12	6.67	0.53			
OPFs/E	27.38	1.97	7.36	0.53			
OPFs/Zn	27.69	2.13	6.98	0.55			
OPFs/C/E/Zn	26.53	1.58	6.60	0.23			
**BC**							
	**SOD**	**CAT**	**GPx**	**MDA**	**NO**	**H_2_O_2_**	**O_2_**
OPFs	22.81	1.40	4.99	0.65	2.52	2.49	1.18
OPFs/C	16.46	1.07	4.71	0.85	2.25	1.91	1.24
OPFs/E	18.76	1.13	5.15	0.78	2.66	1.26	1.25
OPFs/Zn	27.04	2.44	7.24	0.75	2.88	1.25	1.19
OPFs/C/E/Zn	31.20	2.46	7.51	0.44	2.47	1.38	1.02

Heatmap colors indicate the expression level: low—green, medium—yellow, high—red. OPFs: control 6 g kg^−1^ supplementation of orange peel fragments; OPFs/C: 6 g kg ^−1^ supplementation of orange peel fragments and 1.8 g kg^−1^ of vitamin C; OPFs/E: 6 g kg^−1^ supplementation of orange peel fragments and 0.4 g kg^−1^ of vitamin E; OPFs/Zn: 6 g kg^−1^ supplementation of orange peel fragments and 0.2 g kg^−1^ of zinc; OPFs/C/E/Zn: 0.6 g kg^−1^ supplementation of orange peel fragments, 1.8 g kg^−1^ of vitamin C, 0.4 g kg^−1^ of vitamin E, and 0.2 g kg^−1^ of zinc. SOD: superoxide dismutase activity; CAT: catalase activity; GPx: glutathione peroxidase activity; MDA: malondialdehyde concentration; NO: nitric oxide; H_2_O_2_: hydrogen peroxide; O_2_^−^: anion superoxide.

## Data Availability

The data presented in this study are available on request from the corresponding author.

## Abbreviations

OPFs	orange peel fragments
C	vitamin C
E	vitamin E
Zn	zinc
HDOIS	heat/dissolved oxygen-induced stress
TIS	transport-induced stress
BC	bacterial challenge
